# Highly Efficient and Sustainable Spent Mushroom Waste Adsorbent Based on Surfactant Modification for the Removal of Toxic Dyes

**DOI:** 10.3390/ijerph15071421

**Published:** 2018-07-05

**Authors:** Ahmad Alhujaily, Hongbo Yu, Xiaoyu Zhang, Fuying Ma

**Affiliations:** 1 Key Laboratory of Molecular Biophysics of MOE, College of Life Science and Technology, Huazhong University of Science and Technology, Wuhan 430074, China; drahmad@hust.edu.cn (A.A.); yuhongbo@hust.edu.cn (H.Y.); zhangxiaoyu@hust.edu.cn (X.Z.); 2 Biology Department, College of Science, Taibah University, Al Madinah Al Munawarah 41411, Saudi Arabia

**Keywords:** spent mushroom waste, effective adsorbent, surface modification, adsorption, wastewater

## Abstract

The treatment of wastewater always demands eco-friendly and cost-efficient adsorbents. In this paper, spent mushroom waste (SMW) was modified by a cationic surfactant (cetyltrimethylammonium bromide, CTAB) to eliminate toxic dyes. A characterization of adsorbents confirmed that CTAB was successfully embedded into the SMW structure. The spent mushroom waste, modified by CTAB (SMWC), exhibited an excellent adsorption capacity of 249.57 mg·g^−1^, 338.67 mg·g^−1^, and 265.01 mg·g^−1^ for the Direct red 5B (DR5B), Direct blue 71 (DB71), and Reactive black (RB5) dyes, respectively. Batch experiments indicated that the dye adsorption of SMWC depended mainly on pH, dye concentration, temperature, and ionic strength. The adsorption isotherm could be fitted to the Langmuir model and described by the pseudo-second-order kinetic model. The dye adsorption mechanism was dominated mostly by the chemosorption of the dyes and the SMWC surface. Thermodynamic parameters showed that the adsorption was endothermic and spontaneous. SMWC could successfully remove over 90% of dyes from various water samples. This can be considered a feasible waste resource utility, since it meets both the ecological and the economic requirements for auspicious industrial applications.

## 1. Introduction

The risk of water pollution is rapidly increasing in the modern industrialized world because tons of industrial, municipal, and other types of waste are being released into bodies of water. Industrial waste contains different types of organic dyes and toxins [1]. Generally, synthetic dyes contain aromatic compounds consisting of different functional groups that can be categorized into cationic (basic), anionic (direct, acid, and reactive), and nonionic (disperse) [2]. Dyes released into environmental waters can be mutagenic, carcinogenic, and toxic to aquatic life [2], but they are difficult to remove because of their high solubility. Therefore, the removal of dyes from wastewater to avoid severe toxicity to aquatic life is one of the most pressing scientific concerns. So far, several techniques have been employed to combat this issue, including nanofiltration [3], cloud point extraction [4], ozonation [5], flocculation [6], photo-degradation [7], oxidation [8], coagulation [9], and microbial degradation [10]. However, various inadequacies, such as high operational costs, inefficiency, and generation of toxic byproducts, hinder the applicability of these techniques for wastewater treatment [11].

Adsorption is considered a highly attractive method due to its low cost, simple design, recyclability, and high removal efficiency. Several studies have been carried out to develop various adsorbents, such as activated carbon [12,13,14], metal organic frameworks [15], polymers [16], and zeolites [17], but the high cost and environmental toxicity of these adsorbents limit the range of their applicability. Therefore, there is an urgent need for alternative adsorbents that are environmentally friendly, inexpensive, and widely available.

Tons of spent mushroom waste (SMW) are commercially produced worldwide every year. Spent mushroom waste is a residual compost waste generated by the mushroom production industry. Generally, 1 kg of mushrooms yields 5 kg of SMW waste [18], which, in turn, results in pollution. Spent mushroom waste is rich in natural polymers, such as chitosan, chitin, protein, cellulose, and hemicelluloses. Interestingly, these polymers are rich in carbonyl, hydroxyl, and amide functional groups, which could provide active binding sites for pollutants in the wastewater treatment process. In the recent past, it has been reported that unmodified SMW can be used for dye removal, but it has a limited capability to extract anionic dyes in acidic environments [19,20].

However, the limited capability of SMW for dye removal and its sensitivity to low pH could be overcome by modifying it using certain surfactants. The modification of the SMW surface using cationic surfactants can change the surface properties of SMW from hydrophilic to hydrophobic. A successful surface modification was accomplished through the electrostatic attraction between cationic molecules of cetyltrimethylammonium bromide (CTAB) and the negatively charged surface of SMW. To the best of our knowledge, no information on the use of modified SMW for the removal of toxic dyes can be found in the literature. The study of surface modification with CTAB was investigated using various characterization techniques, including Fourier-transform infrared (FTIR), X-ray diffraction (XRD), thermogravimetric analysis (TGA), scanning electron microscopy (SEM), energy dispersive X-ray spectroscopy (EDX), and X-ray fluorescence (XRF). Characterization results confirmed that CTAB was successfully embedded into the SMW structure. Moreover, the impact of SMW modified by CTAB (SMWC) dosage, temperature, dye dosage, pH, and ionic strength on the adsorption mechanism was also studied.

## 2. Materials and Methods

### 2.1. Materials

Direct Red 5B (DR5B), Direct Blue 71 (DB71), Reactive Black 5 (RB5), acid orange II (Sigma-Aldrich, Shanghai, China), hydrochloric acid, sodium hydroxide, sulphuric acid, chloroform, and CTAB (Sinopharm Chemical Reagent Co., Ltd., Shanghai, China) (Table S1) were all of analytical grade and diluted with purified distilled water according to the prescribed protocols of the manufacturers. The final concentration of the stock dye solutions was 1 g L^−1^. The spent mushroom waste of *Pleurotus ostreatus* cultivation was provided by Huazhong Agricultural University (Wuhan, China).

### 2.2. Preparation and Characterization of Adsorbents

Dried SMW was ground in a blender and then sieved, producing small particles in the range of 0.2 and 0.4 mm. To achieve the SMW modification, 10 g of SMW powder was added to various concentrations (5–60 mmol·L^−1^) of CTAB solution and stirred at room temperature. After 24 h, the mixture was washed several times to remove the CTAB and dried overnight at 60 °C.

The amount of surfactant loaded onto the surface of SMW at different concentrations was evaluated by spectrophotometry [21]. To determine the CTAB concentration, the solution needed to be acidic. In a separating funnel, 25 mL of distilled water, 5 mL of 2 M sulphuric acid (H_2_SO_4_), and 10 mL of chloroform were gently mixed, and approximately 2 mL of 1 M acid orange II was then added to 1 mL of the sample solution. The mixture was shaken vigorously and then left to settle until the chloroform layer was extracted. The chloroform phase was measured at the absorbance of 487 nm using a UV–Vis spectrophotometer (Shimadzu, Kyoto, Japan).

Fourier-transform infrared spectra of the sample were recorded in the wavenumber range of 400–4000 cm^−1^ using a Thermo Nicolet spectrophotometer (Thermo Scientific, Waltham, MA, USA) and the KBr pellet method (IR grade, Merck, Germany). The study of the surface morphological characteristics of SMW and SMWC were investigated using a Quant 200 field emission scanning electron microscope (Philips XL30, Eindhoven, The Netherland). The elemental present on the sample surface was tested by energy-dispersive X-ray spectroscopy (Mahwah, NJ, USA) coupled with SEM. The properties of SMW were studied using X-ray fluorescence (Shimadzu XRF1800, Kyoto, Japan). The crystalline properties of SMW and SMWC were determined using an X-ray diffractometer (PANalytical, Almelo, The Netherlands). The thermal properties of SMW and SMWC were determined using a thermal gravimetric analyzer (Netzsch STA409, Bavaria, Germany).

### 2.3. Dye Adsorption Study of SMWC 

The adsorption experiments were performed in a batch of 100 mL sealed conical flasks containing 50 mL of dye solution. The effect of adsorbent dosage was evaluated by agitating different masses of the adsorbent with an initial dye concentration of 200 ppm of DR5B and 150 ppm of RB5. The effect of pH on the dye adsorption of SMWC was investigated by adding a negligible 0.1 M HCl or 0.1 M NaOH and thus adjusting the pH from 3 to 11.

The pH of the point of zero charges (pH_PZC_) of SMWC was determined following a method reported in [22]. After the dyes were adsorbed onto SMWC, samples were collected periodically and analyzed using a UV–Vis spectrophotometer to measure the absorbance at wavelengths of 540 nm, 590 nm, and 587 nm with DR5B, DB71, and RB5, respectively. For a desorption study, dye-loaded SMWC was added to 10 mL of 0.1 M NaOH and agitated on a rotary shaker for 4 h at room temperature. The adsorbent was collected by centrifugation, then rinsed with water several times and re-used in a recycling study.

All experiments were performed in triplicate, and, finally, the adsorption capacity was determined according to Equation (1):

(1) $$\mathrm{Adsorption}\;\mathrm{capacity}=\frac{\left(C_{0}-{\;C}_{e}\right)V}{m}$$

where *C*_0_ (ppm) is the initial dye concentration, *C_e_* (ppm) is the equilibrium of the dye concentration, *V* (L) is the volume of the dye solution, and *m* (g) is the mass of the adsorbent. The removal efficiency (%) was calculated according to Equation (2):

(2) $$\mathrm{Removal}\mathrm{efficacy}\;\left(\%\right)=\frac{C_{0}-C_{e}}{C_{0}}\times 100$$

### 2.4. Modeling Study

In the kinetic experiment, about 75 mg of SMWC was added to 50 mL of dye solution at a concentration of 200 ppm of DR5B and DB71, and 150 ppm of RB5 was magnetically stirred at 150 rpm for 420 min at 30 °C. The samples were withdrawn at regular time intervals to analyze their residual dye concentration. Five different models were applied to study the dye adsorption behavior: the pseudo-first-order, pseudo-second-order kinetic, Elovich, intraparticle diffusion, and Boyd models (Equations (3)–(7), respectively):

(3) $$q_{t}{=q}_{e}1-exp\left(k_{f}.t\right)$$

(4) $$q_{t}=\frac{k_{s}{\;q}_{e}^{2}\;t}{1{+q}_{e}{\;k}_{s}\;t}$$

(5) $$q_{t}=\frac{1}{\beta }In(\alpha \beta )+\frac{1}{\beta }lnt$$

(6) $$q_{t}{=k}_{i}t^{1/2}+C$$

(7) $$B_{t}=-0.4977-In\;(1-F)$$

where *q_e_* (mg·g^−1^) and *q_t_* (mg·g^−1^) are the amount of dye adsorbed by SMWC at equilibrium and time *t*, respectively. *k_f_* (min^−1^) is the pseudo-first-order rate constant, *k_s_* (g·mg^−1^·min^−1^) is the pseudo-second-order rate constant, α (mg·g^−1^·min^−1^) is the initial adsorption rate constant, β (g·mg^−1^) is the constants relating to the extent of the surface covered and the chemisorption activation energy, respectively, and *k_i_* (mg·g^−1^·min^−1/2^) is the intraparticle diffusion model constant. *F* = *q_t_*/*q_e_* refers to the fractional achievement of equilibrium at time *t*.

The adsorption isotherms are significant for understanding the interaction between the adsorbent and the adsorbate [23]. The experiment was performed at the initial dye concentrations of 50–2200 ppm. The dye solution was stirred at 150 rpm for 420 min at 30 °C, and the residual dye concentration of the sample was analyzed. Four different models were applied to determine the dye adsorption behavior: the Langmuir, Freundlich, Redlich–Peterson, and Dubinin–Radushkevich models, which are as follows (Equations (8)–(13)):

(8) $$q_{e}=\frac{q_{m\;\;}K_{L}C_{e}\;}{{1+K}_{L\;}\;C_{e}}$$

(9) $$q_{e}{=K}_{n}C_{e}^{\left(1/n\right)}$$

(10) $$q_{e}=\frac{K_{r}C_{e}\;}{1+a_{r}\;C_{e}^{g}}$$

(11) $$q_{e}=q_{m\;}\exp\left(-B\varepsilon^{2}\right)$$

(12) $$\varepsilon =\;RT\;In\left(1+\frac{1}{C_{e}}\right)$$

(13) $$E=\;\frac{1}{\sqrt{2B}}$$

where *q_m_* (mg·g^−1^) is the theoretical maximum saturated capacity of the adsorbent; *K_L_* (L/mg) is the Langmuir isotherm constant; *K_n_* ((mg/g)/(L/mg)^1/n^) is the Freundlich isotherm constant; *n* is a Freundlich intensity parameter; *Kr* (L/g) and *a_r_* ((L/mg)g) are the Redlich–Peterson isotherm constants; *g* is the isotherm exponent that lies between 0 and 1; *B* (mol^2^/kJ^2^) is the Dubinin–Radushkevich constant; ε (J/mol) is the Polanyi potential; *R* (8.314 J/mol K) is the gas universal constant; *T* (*K*) is the absolute temperature; and *E* (kJ/mol) is the mean adsorption energy.

### 2.5. Application of SMWC for the Removal of Dyes from Urban and Industrial Water

Different real water samples, including seawater (Shenzhen, Guangdong), lake water (Eastern Lake, Wuhan, Hubei), industrial wastewater (Wuhan, Hubei), and tap water (Wuhan, Hubei), with 75 mg of SMWC and 200 ppm of DR5B, DB71, and 150 ppm of RB5, respectively, were tested in 50 mL of dye solution at 150 rpm for 420 min at 30 °C to determine the adsorption efficiency of SMWC.

## 3. Results and Discussion

### 3.1. Characterization of SMW and SMWC

**Figure 1:**
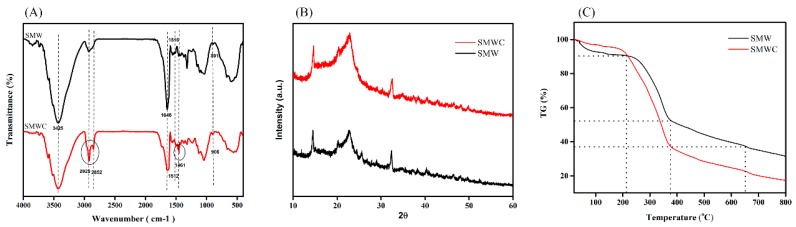
FTIR spectra of spent mushroom waste (SMW) and SMW modified by cetyltrimethylammonium bromide (SMWC) (**A**); X-ray diffraction patterns of SMW and SMWC (**B**) and the TGA of SMW and SMWC (**C**).

The FTIR, XRD, TGA, XRF, and SEM-EDX analyses were performed to evaluate the surface modification of SMW with CTAB. The Fourier-transform infrared spectrum (Figure 1A) of the SMW adsorbent was characteristic for a lignocellulose material containing natural polymeric compounds, such as carboxylic acids, alcohols, and phenol [24,25]. The broad band at approximately 3425 cm^−1^ corresponded to hydroxyl groups on the SMW surface, which indicated the presence of cellulose and lignin. Compared to the spectrum of SMW, the spectrum of SMWC exhibits highly intense peaks at 2925 cm^−1^, 2852 cm^−1^, and 1461 cm^−1^, along with prominent aliphatic groups, such as CH, CH_2_, and CH_3_, which correspond with the functional groups of CTAB. However, the decreased band intensity at 1646 cm^−1^ could be attributed to the water present in the adsorbent, which indicates a reduced water content due to the replacement of hydrated cations with surfactant cations [26]. Furthermore, changes in the peaks at 905 cm^−1^ (C–O) and 2852 cm^−1^ (aliphatic C–H), and the shift of the peak at 1516 cm^−1^ (aromatic C=C) to 1512 cm^−1^, in SMWC suggested that π–π interaction was involved in the surface modification [27].

The XRD analysis was used to explore the crystallinity of SMW as well as the modification of CTAB (Figure 1B). Generally, SMW and SMWC had similar peak patterns. Peaks, typical of lignocellulose, appeared at 2*θ* = 22° and 2*θ* = 14°, and the 2*θ*° closer to 22° showed crystallinity, while the 2*θ*° closer to 16° showed an amorphous structure [28,29]. The intensity of crystalline peaks typically increased in SMWC. The low intensity of unmodified SMW was attributed to amorphous cellulose, hemicelluloses, and lignin. The increase in crystallinity after the modification with CTAB could be related to the attachment of the bulky CTAB structure to the SMW surface [28].

Thermogravimetric analysis was used to further compare the thermal stability of SMW and SMWC under a nitrogen atmosphere. As shown in Figure 1C, a decrease in mass occurred in three stages at temperatures of 30–250 °C, 250–400 °C, and 400–650 °C, which correspond to dehydration, depolymerization, and decomposition, respectively. In the first phase (30–250 °C), the decrease in mass could be attributed to the removal of water molecules from the absorbent and the decomposition of substances, such as proteins and low molecular weight organic compounds. In the second phase (250–400 °C), the sharp decrease in mass was due to the degradation of the main hemicellulose skeleton in SMW and SMWC. The third phase resulted in further decomposition of cellulose and lignin components in SMW and SMWC [30,31,32]. In the temperature range of 200–650 °C, the mass decreased by 37% and 22% in SMW and SMWC, respectively. The more pronounced decrease in the mass of SMWC, as compared with that of SMW, could be attributed to the degradation of additional organic content attached to the SMWC surface due to the CTAB modification. 

**Figure 2:**
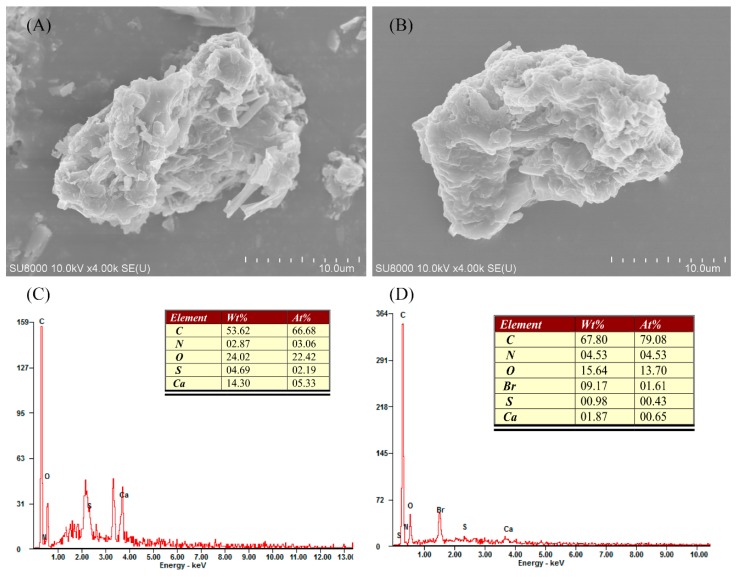
SEM and EDX analysis of SMW (**A**,**C**); and SMWC (**B**,**D**).

Scanning electron micrographs showed the external surface of SMW and SMWC. It can be found that both SMW and SMWC had a defined pore structure (Figure 2A). After the CTAB modification, the SMWC surface was more uniform and the layered stacks became smooth, which might be due to the incorporation of surfactant in the surface of SMW (Figure 2B). Energy dispersive X-ray analysis showed that SMWC was mainly composed of carbon, nitrogen, oxygen, calcium, and sulfur. The number of calcium ions was much lower after the CTAB modification, suggesting the exchange of intercalated surfactant cations [33]. In addition, bromine was present on the SMWC surface and absent from the SMW surface (Figure 2C,D), suggesting that a fraction of molecules were hexadecyltrimethylammonium bromide, which can be arranged using a mode of bimolecular association [34]. The inorganic chemical composition of SMW and SMWC, determined by XRF, was as follows: SMW—11.63% of SiO_2_, 61.29% of CaO, 10.03% of SO_3_, 5.74% of Fe_2_O_3_, 6.19% of P_2_O_5_, 0.76% of MnO, 0.47% of Al_2_O_3_, 3.77% of K_2_O, and 0.12% of ZnO; SMWC—12.24% of SiO_2_, 61.75% of CaO, 6.35% of SO_3_, 5.83% of Fe_2_O_3_, 5.80% of P_2_O_5_, 3.96% of MnO, 1.95% of Al_2_O_3_, 0.50% of K_2_O, 0.36% of ZnO, and 1.26% of Br.

### 3.2. Effect of Surfactant Concentration on the SMW Surface and Its Impact on the Removal of Dyes

**Figure 3:**
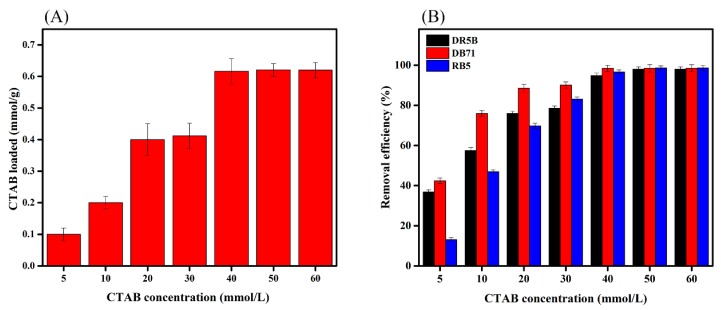
The CTAB concentration on SMW surface (**A**) and the influence of CTAB concentration on dye removal (**B**).

The initial CTAB concentration had a significant impact on the surfactant loaded onto the SMW surface. The CTAB loaded onto the SMW surface improved with increasing surfactant concentrations and reached nearly 0.60 mmol·g^−1^ (Figure 3A). The attachment of CTAB to the surface of SMW may be due to the electrostatic attraction between the cationic charged parts of CTAB and the negatively charged sites of SMW. Initially, when the concentration of surfactant was lower than the critical micelle concentration, surfactant molecules were available as monomers to be adsorbed on the SMW surface more easily [35]. When the surfactant concentration exceeded the critical micelle concentration due to hydrophobic interactions or surfactant micelle formation and aggregation over the surface, this inhibited the further addition of CTAB to the SMW [35,36]. Furthermore, the EDX results confirmed a decrease in Ca ions, which suggested that the cationic surfactant might have replaced some Ca ions through an ion exchange in the SMW matrix [37].

An enhanced adsorption capacity, after the modification with CTAB, contributed to hydrophobic interactions between the dyes and hydrophobic tails of the surfactant. The positively charged head of CTAB enhanced electrostatic interactions between the adsorbent surface and anionic dyes. The removal efficiency of all dyes linearly increased as the concentration of surfactant loaded onto the SMW increased to 50 mmol L^−1^ (Figure 3B). However, when the CTAB concentration exceeded 40 mmol·L^−1^, dye removal reached a plateau, which could be attributed to a maximum saturation of CTAB on the SMW surface.

### 3.3. Effects of pH and Ionic Strength on the Dye Adsorption Capacity of SMWC

**Figure 4:**
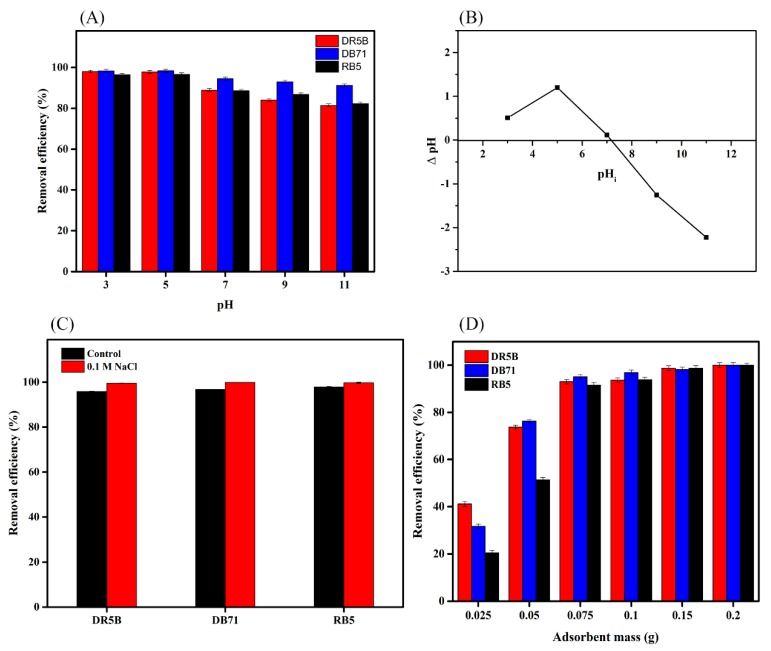
Effect of (**A**) pH; (**B**) the point of zero charge of SMWC; (**C**) ionic strength; and (**D**) adsorbent dosage on dye removal.

The surface properties of the adsorbent are influenced by pH and ionic strength, which are therefore important parameters in adsorption. The influence of pH, which initially ranged from 3.0 to 11.0, on the removal efficacy of different dyes in SMWC is shown in Figure 4A. The removal efficiency of DR5B, DB71, and RB5 increased from 3.0 to 5.0 and then slightly decreased as the pH decreased from 7.0 to 11.0. The maximum removal efficiencies of DR5B, DB71, and RB5, with a pH ranging from 3.0 to 5.0, were 97.9%, 98.3%, and 96.4%, respectively. The tolerance of the dyes to pH was generally associated with the surface charge of the adsorbent at various pH values. This is consistent with the result that the pH_PZC_ of SMWC was about 7.2 (Figure 4B), suggesting that the surface charge lower than 7.2 was more protonated, which could be attributed to the electrostatic attraction between the electropositive adsorbent surface and anionic dyes. However, in this case, the high removal efficiency of the dyes on the adsorbent remained at pH 9 due to the chemical interactions between the dyes and SMWC. This can be concluded from the FTIR spectra results after adsorption. 

We further explored the influence of ionic strength on the dye removal efficiency of SMWC (Figure 4C). The result indicated that the removal efficiency increased upon the addition of 0.1 mol·L^−1^ NaCl into the solution. The increase in removal efficiency upon the addition of NaCl could be attributed to the increase in dye dimerization [38]. Furthermore, NaCl ions forced the dye molecules to aggregate, leading to an increase in the extent of adsorption on the SMWC surface [39].

### 3.4. Effect of the Adsorbent Dose on Dye Removal

The increased availability of active sites for the interaction of adsorbents and dyes was important for the efficiency of the dye removal process [40]. We investigated the influence of adsorbent dosage, ranging from 25 to 200 mg, on dye removal. The removal efficiency increased dramatically as the adsorbent dosage increased, which was due to an increased availability of active sites for interacting with dyes (Figure 4D). However, the removal efficiency did not increase at the higher adsorbent dosage because the latter had already reached the saturation level (Figure 4D).

### 3.5. Adsorption Kinetics

**Figure 5:**
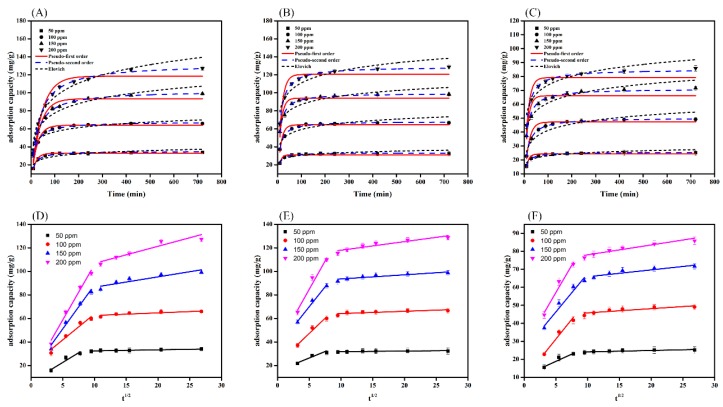
Adsorption kinetic analysis with different concentrations of Direct red 5B (DR5B) (**A**,**D**), Direct blue 71 (DB71) (**B**,**E**), and Reactive black 5 (RB5) (**C**,**F**) and using different kinetic models.

The adsorption capacity of all dyes (DR5B, DB71, and RB5) initially increased and then remained constant after reaching equilibrium (Figure 5A–C). Further, the contact time to reaching equilibrium also increased with the increase in the initial dye concentration. The initial rapid increase in adsorption capacity was attributed to the availability of a higher number of active binding sites on the SMWC surface [41]. Another reason might be an increase in mass driving force, which leads to a transfer of dye molecules from the solution to the SMWC surface.

The study of experimental kinetics data was evaluated using pseudo-first, pseudo-second, Elovich, and intraparticle diffusion. The results obtained after applying these models are summarized in Table S2. The correlation coefficient *R*^2^ of the pseudo-second-order model ranged from 0.989 to 0.999 for DR5B, DB71, and RB5, indicating that the *R*^2^ values of second-order model were higher than those of the other models (Table S2). Therefore, the adsorption kinetics highly resembled the pseudo-second-order model, suggesting the chemisorption [42,43] (Figure 5A–C). Further, these results were in accordance with the FTIR findings, which revealed the incorporation of bonds in dyes into SMWC surface after adsorption (discussed in mechanism section). As reflected in Table S2, the values of the pseudo-second-order rate constant (*k_s_*) gradually decreased in all dyes as the initial concentration increased. This behavior might stem from the increased driving force at higher dye concentrations [44].

Additionally, the adsorption data were fitted to the intraparticle diffusion model (Figure 5D–F). The intraparticle diffusion model is shown in Figure 5D–F. This suggested that both diffusion and intraparticle diffusion processes took place simultaneously during the adsorption of the dyes. Moreover, the multilinearity demonstrated that the adsorption was not only controlled by intraparticle diffusion [45]. Further, to predict the actual rate-controlling step involved in the adsorption process, we applied the Boyd kinetic model. As shown in Figure S1, the plot of the Boyd model does not pass through the origin, suggesting that the diffusion process might be the rate-controlling step of the adsorption process [45].

### 3.6. Adsorption Isotherms

The equilibrium adsorption isotherms are significant for the adsorption process, in which adsorbates are absorbed. The adsorption isotherms obtained for dyes adsorption on SMWC for DR5B, DB71, and RB5 show that the adsorption capacity increased as the initial dye concentrations increased from 50 to 2200 ppm and gradually reached the maximum adsorption capacity at 228 mg g^−1^, 289 mg g^−1^, and 219 mg g^−1^ for DR5B, DB71, and RB5, respectively. This is attributed to the increased bulk flow between the dyes and SMWC, which increased the availability of adsorption sites on the surface of the SMWC.

**Figure 6:**
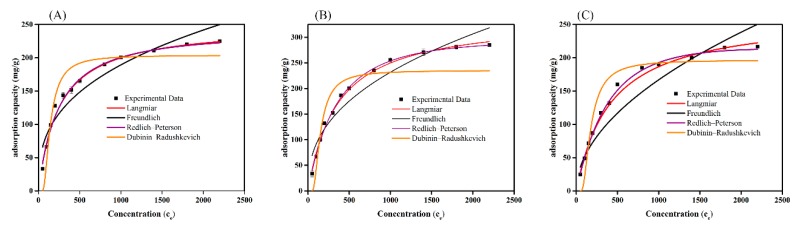
Adsorption isotherm model of (**A**) DR5B, (**B**) DB71, and (**C**) RB5 on SMWC.

Further, various isotherm models were used to investigate the adsorption behavior as well as the maximum experimental capacity (Figure 6A–C). The evaluated parameters, along with the corresponding regression coefficients, are summarized in Table S3. On the basis of the regression coefficients (Table S3), the dye adsorption can be described by the Langmuir model (*R*^2^ > 0.99). Based on the Langmuir isotherm model, the theoretical maximum monolayer adsorption capacities of DR5B, DB71, and RB5 were found to be 249.57 mg g^−1^, 338.67 mg g^−1^, and 265.01 mg g^−1^, respectively. From (Table 1), it is apparent that SMWC has a superb adsorption capacity compared to the well-known biomass-, lignin-, or fly-ash-based adsorbents. Additionally, the Dubinin−Radushkevich model was used to free energy from adsorption on nouniform surfaces. As shown in Table S3, the E values of 14.43 kJ mol^−1^, 13.65 kJ mol^−1^, and 11.78 kJ mol^−1^ were found for DR5B, DB71, and RB5, respectively. The *E* values were in the range of 8–16 kJ mol^−1^, suggesting the adsorption process involved the chemosorption [46,47]. The Redlich–Peterson isotherm was applied, which incorporates the Langmuir and Freundlich isotherm models. When *g* = 1, the Redlich–Peterson isotherm was reduced to the Langmuir equation, and when *g* = 0, the isotherm was reduced to the Henry’s isotherm [48]. It can be seen that the value of *g* is close to 1 for DR5B, DB71, and RB5, which indicates that the adsorption process is close to the Langmuir equation. 

### 3.7. Temperature Effect on Dye Removal and Thermodynamic Analysis

**Figure 7:**
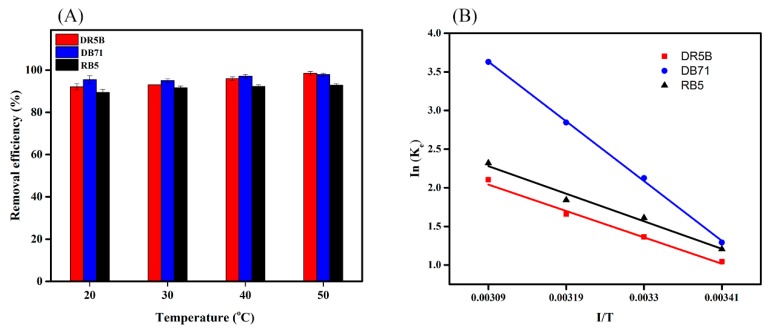
Effect of temperature on dye removal (**A**) and thermodynamic study (**B**).

Temperature has a significant effect on the adsorption process. The removal efficacy of DR5B increased from 92% to 98% when the temperature increased from 20 to 50 °C, and the same trend was present in DB71 and RB5 (Figure 7A). It is well-known that an increase in temperature leads to an increase in the diffusion rate of dye molecules passing through the external boundary layer into the inner pores of the adsorbent particles [56]. Further, an increase in temperature may enhance the fluidity of dye molecules, providing sufficient energy to promote the interaction between the dye molecules and adsorption sites. An increase in temperature results in the expansion of the internal structure of the adsorbent, allowing more dye molecules to pass through [57].

Thermodynamic properties were investigated to determine whether the process is spontaneous or not and also to understand the sorption behavior. Thermodynamic parameters, including the change in entropy (Δ*S°*), Gibbs free energy (Δ*G°*), and enthalpy (Δ*H°*) in the dye adsorption, were determined according to the following equations [58]:

(14) $$K_{d}=\frac{C_{ad,e}}{C_{e}}$$

(15) $$\Delta G^{o}=-{RTlnK}_{d}$$

(16) $$lnK_{d}=\frac{\Delta S{}^{\circ}}{R}-\frac{\Delta H{}^{\circ}}{RT}$$

where *R* (8.3143 J/mol K) is the gas constant; *K_d_* is the equilibrium coefficient of the adsorption; *C_ad,e_* (mg L^−1^) is the concentration of adsorbed dyes at equilibrium; and *T* (*K*) is the temperature. The values of Δ*H°* and Δ*S°* can be estimated from the slopes and intercepts by plotting a graph of *InK_d_* against 1/*T* (Figure 7B). The negative value for Δ*G°* shows that the adsorption of dye molecules on SMWC was spontaneous and favorable. As detailed in Table S4, Δ*G°* increased as the temperature increased and was negative at all temperatures. The negative values of Δ*G°* suggest that there were electrostatic interactions between the dyes and SMWC. Higher values of Δ*G°* at a high temperature indicates that there was an endothermic process, which is further confirmed by the positive Δ*H°* value. The Δ*H°* value suggests that Van der Waals interactions, dipole bonds, electrostatic interactions, and hydrophobic interactions may have also contributed to the adsorption process [59].

### 3.8. Adsorption Mechanism

**Figure 8:**
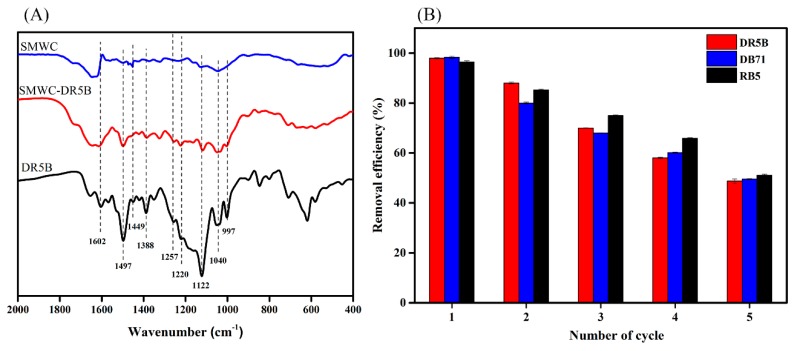
FTIR spectra of SMWC, SMWC-DR5B, and DR5B alone (**A**); Dye adsorption of SMWC at five regeneration cycles (**B**).

Both the physical and chemical adsorption mechanism may be considered during the adsorption of dyes on SMWC. Initially, the pore structure of SMWC, as suggested by SEM analysis, definitely offered easily accessible sites for dye molecules to contribute to physical adsorption on the surface. On the other hand, according to our kinetics and isotherm experiments, the removal of dyes was dominated by chemical adsorption rather than physical adsorption. These findings were also supported by the influence of pH and point of zero charges (pH_PZC_), which also demonstrated the dominant chemical adsorption mechanism. In order to get a clear picture of adsorption mechanism, we conducted FTIR analysis for DR5B dye alone and SMWC before and after adsorption of DR5B to investigate the structural changes (Figure 8A). As shown, DR5B demonstrated FTIR bands for N-H (1604 cm^−1^), aromatic C=H (1497 cm^−1^ and 1449 cm^−1^), C–O (1388 cm^−1^), S=O (1254 cm^−1^, 1122 cm^−1^, and 1040 cm^−1^), and C–O (997 cm^−1^) [26,60]. After the adsorption of DR5B dye over SMWC, all of these bands consistently appeared, which suggested the binding of dyes to SMWC through these functional groups. In addition, the new band for thio groups (S=O) at 1040 cm^−1^ confirmed the bonding through electrostatic attraction between the anionic SO_3_^−^ group of dye molecules and the positively charged quaternary ammonium group on the SMWC surface. Additionally, the adsorption reaction could change the molecular and crystal structure of the adsorbent. Therefore, we conducted XRD analysis for SMWC before and after adsorption to understand the changes in molecular and crystal structure of the adsorbent. The XRD patterns of dye-loaded adsorbents are presented in Figure S2. As shown, the peak intensity increased after adsorption without additional peaks, which suggested that no significant changes in the structure of the adsorbent occurred after adsorption. Furthermore, these results also confirmed the dyes’ adsorption on the external surface of adsorbent. 

### 3.9. Dye Removal from Different Water Samples

We tested the efficiency of SMWC as an adsorbent for dye removal in different water samples, including tap water, industrial wastewater, lake water, and seawater (Table S5). The dye removal efficiency reached 95–99% in tap water and seawater. However, the dye removal efficiency of SMWC in industrial wastewater was slightly lower (80–91%), which may be due to its pH. Compared with the pH of industrial wastewater, which was over 8.10, the pH of tap water and seawater ranged from 6.5 to 7.5, which was a more suitable range for adsorption by SMWC. The higher ionic strength of seawater and tap water might be conducive to an increase in dye adsorption efficiency.

### 3.10. Recyclability Study

Adsorbent recycling after the removal of pollutants is important, both economically and environmentally. The restoration of the SMWC was achieved using a 0.1 M NaOH solution. As shown in Figure 8B, the dye removal efficiency of DR5B, DB71, and RB5 was more than 70%, even after the dyes had been recycled three times. This result demonstrates that SMWC can be effectively recycled and reused for the adsorption of dyes, which is the most important requirement for an absorbent.

## 4. Conclusions 

In summary, we present a cost-effective, highly efficient, and eco-friendly SMWC adsorbent for dye removal. The FTIR, XRD, TGA, SEM-EDX, and XRF results confirmed successful modification of SMW with CTAB. The SMWC adsorbent was applied for adsorption of various anionic dyes including DR5B, DB71, and, RB5. Various parameters such as adsorbent amount, dye concentration, effect of temperature, influence of pH and ionic strength, etc., were evaluated in detail. Furthermore, the adsorption isotherm and kinetic studies suggested best fitting for the Langmuir model and pseudo-second-order kinetic model, thus revealing that the adsorption process was essentially controlled by the chemisorption mechanism. These findings were also supported by the FTIR studies. Further, the influence of the initial pH solution suggested a broad pH window for turning SMWC into an effective adsorbent for the elimination of toxic dyes from wastewater.

## Figures and Tables

**Table 1:** Comparison of the maximum adsorption capacities of some anionic dyes on various adsorbents.

Adsorbent	*C*_0_ (ppm)	Adsorbent Dose (g)	Adsorbate	*Q*_m_ (mg·g^−1^)	Reference
pine cone acid treated	10–60	0.02	Congo Red	40.19	[49]
wheat shells	50–250	0.5	Direct blue 71	46.30	[50]
Fungal biomass	10-400	0.2	Reactive Black 5	106	[51]
Chitosan beads	30–400	0.3	Reactive Red 120	129.9	[52]
Na-Bentonite	50–1000	5	Congo Red	36	[53]
Cetylpyridinium-bentonite	10–70	0.01	Reactive Red 120	81.97	[54]
Sunflower seed shells	15–50	5	Reactive Black 5	1.10	[55]
SMWC	50–2200	0.075	Direct Red 5B	249.57	in this study
SMWC	50–2200	0.075	Direct blue 71	338.67	in this study
SMWC	50–2200	0.075	Reactive Black 5	265.01	in this study

## Supplementary Materials

The following are available online at http://www.mdpi.com/1660-4601/15/7/1421/s1: Table S1: General characteristics of CTAB and the dyes used in this study; Table S2: Kinetic model parameters for dye adsorption on the SMWC; Table S3: Values of different isotherm parameters in dye adsorption on SMWC; Table S4: Thermodynamic parameters of dye adsorption on SMWC; Table S5: Adsorption of dyes from real water samples on SMWC; Figure S1. Boyd kinetic model for different concentration of DR5B (A), DB71 (B), and RB5 (C); Figure S2. XRD of SMWC absorbing DR5B, DB71, and RB5.
