# Supplementary material for: Highly Efficient and Sustainable Spent Mushroom Waste Adsorbent Based on Surfactant Modification for the Removal of Toxic Dyes
Source: Int J Environ Res Public Health. 2018 Jul 5;15(7):1421. doi: 10.3390/ijerph15071421 (PMC6068660; doi:10.3390/ijerph15071421)
Supplement: Supplementary file 1 [file ijerph-15-01421-s001.pdf]

International Journal of
Environmental Research and Public Health

MDPI

Supplementary Materials

Table S1: General characteristics of CTAB and the dyes used in this study.

Compound	Molecular formula	Structural formula	Molecular weight
CTAB	$C_{19}H_{42}BrN$	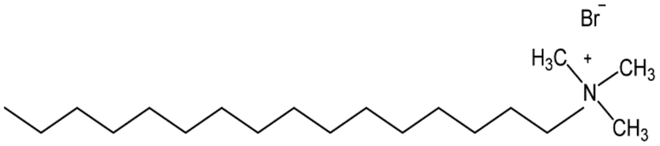	364.45
DR5B	$C_{29}H_{19}N_5Na_2O_8S_2$	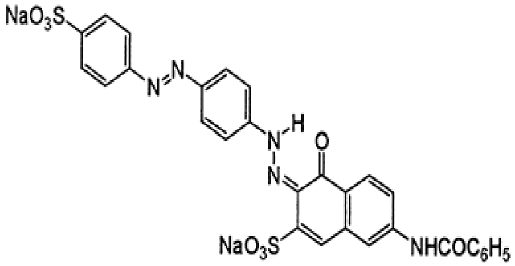	675.6
DB71	$C_{40}H_{23}N_7Na_4O_{13}S_4$	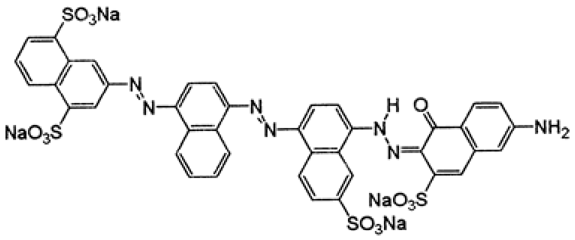	1029.87
RB5	$C_{26}H_{21}N_5Na_4O_{19}S_6$	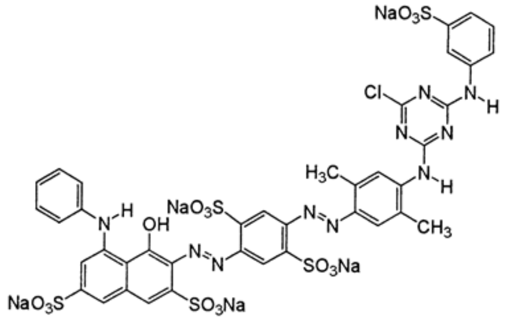	991.82

Table S2: Kinetic Model Parameters for dye adsorption on the SMWC

	DR5B				DB71				RB5			
Concentration (ppm)	50	100	150	200	50	100	150	200	50	100	150	200
$q_{e,exp}$ (mg g^{-1})	33.83	65.87	99.12	127.37	32.33	66.57	98.63	128.73	25.10	49.08	71.61	85.86
Pseudo-first-order												
$q_{e,col}$ (mg g^{-1})	32.82	63.91	93.45	118.52	31.14	64.48	94.14	120.49	24.27	47.28	66.14	79.18
k_f (min^{-1})	0.070	0.042	0.028	0.023	0.114	0.075	0.077	0.0643	0.094	0.058	0.075	0.068
R^2	0.979	0.916	0.936	0.944	0.926	0.930	0.874	0.874	0.906	0.963	0.918	0.881
Pseudo-second-order												
$q_{e,col}$ (mg g^{-1})	34.99	67.79	102.41	131.24	33.17	68.22	99.56	129.12	25.42	50.25	71.14	85.09
k_s (g mg^{-1} min^{-1})	0.0026	0.0011	0.0004	0.0002	0.0058	0.0017	0.0012	0.0007	0.0061	0.0016	0.0017	0.0012
R^2	0.989	0.991	0.996	0.995	0.997	0.995	0.992	0.997	0.999	0.999	0.991	0.995
Elovich												
α (mg g^{-1} min^{-1})	61.81	307.60	25.52	15.85	1007.40	276.43	800.63	404.14	696.82	31.33	128.41	269.15
β (mg g^{-1} min^{-1})	0.25	0.15	0.07	0.045	0.34	0.14	0.10	0.07	0.45	0.15	0.12	0.11
R^2	0.784	0.799	0.930	0.960	0.814	0.838	0.853	0.858	0.804	0.900	0.904	0.890
Intraparticle diffusion												
$k_{i,1}$ (mg g^{-1} $min^{-1/2}$)	6.22	19.78	13.81	11.73	15.41	21.45	36.11	36.11	10.83	9.22	25.03	26.81
C_1 (mg g^{-1})	3.28	4.40	7.47	9.40	2.19	5.16	6.77	9.61	1.60	4.40	4.26	3.16
R^2	0.968	0.976	0.990	0.993	0.921	0.977	0.989	0.988	0.978	0.975	0.970	0.975
$k_{i,2}$ (mg g^{-1} $min^{-1/2}$)	31.53	60.30	77.80	92.33	31.25	62.18	90.07	110.68	23.30	43.46	62.37	72.54
C_2 (mg g^{-1})	0.090	0.221	0.875	1.44	0.051	0.194	0.350	0.728	0.079	0.233	1.66	0.548
R^2	0.889	0.760	0.845	0.903	0.547	0.631	0.833	0.901	0.764	0.798	0.880	0.903

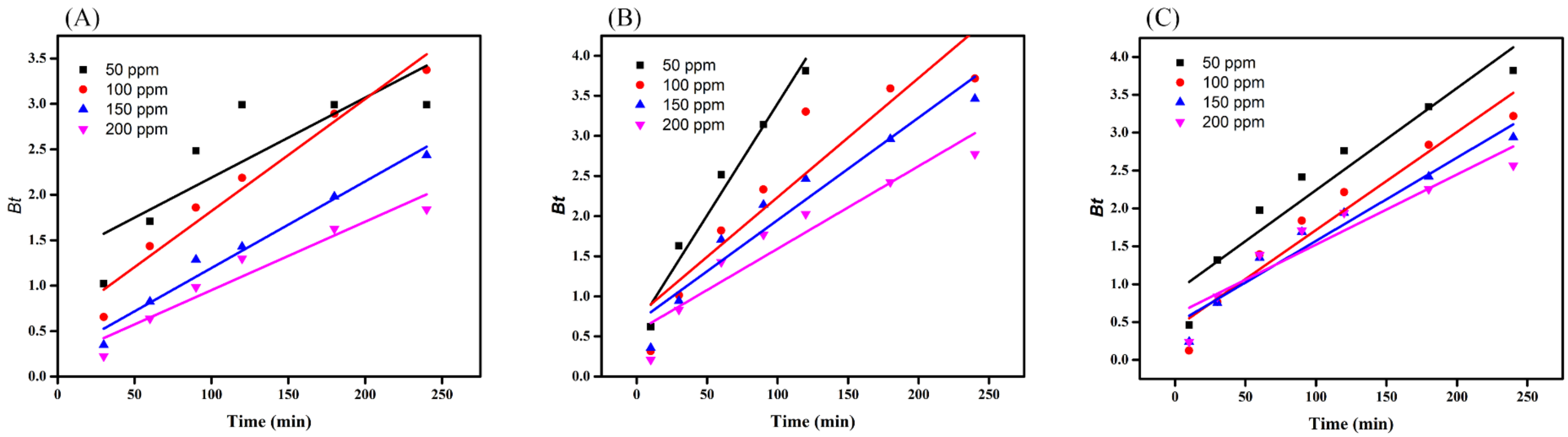

Figure S1. Boyd kinetic model for different concentration of DRB5 (A), DB71 (B), and RB5 (C).

Table S3: Values of different isotherm parameters in dye adsorption on SMWC.

	Langmuir isotherm				Freundlich isotherm		
	DR5B	DB71	RB5		DR5B	DB71	RB5
q_m	249.57	338.67	265.01	K_n	16.61	14.30	4.77
K_L	0.0042	0.0028	0.0023	n	2.83	2.48	1.943
R^2	0.992	0.994	0.995	R^2	0.912	0.911	0.947
	Redlich–Peterson isotherm				Dubinin–Radushkevich isotherm		
K_r	0.962	0.843	0.551	q_m	203.92	235.44	196.69
a_r	0.003	0.0012	0.0005	B	0.0024	0.0027	0.0036
g	1.02	1.09848	1.176	E	14.43	13.66	11.785
R^2	0.993	0.994	0.998	R^2	0.926	0.855	0.899

Table S4. Thermodynamic parameters of dye adsorption on SMWC.

Temperature (°C)	Thermodynamic parameters		
	ΔG (kJ/mol)	ΔH (kJ/mol)	ΔS (J/mol K)
DR5B			
20	-5.86	54.31	229.58
30	-7.91		
40	-9.59		
50	-13.01		
DB71			
20	-7.25	123.44	502.66
30	-12.33		
40	-17.04		
50	-22.44		
RB5			
20	-4.97	70.68	293.21
30	-10.06		
40	-11.03		
50	-14.34		

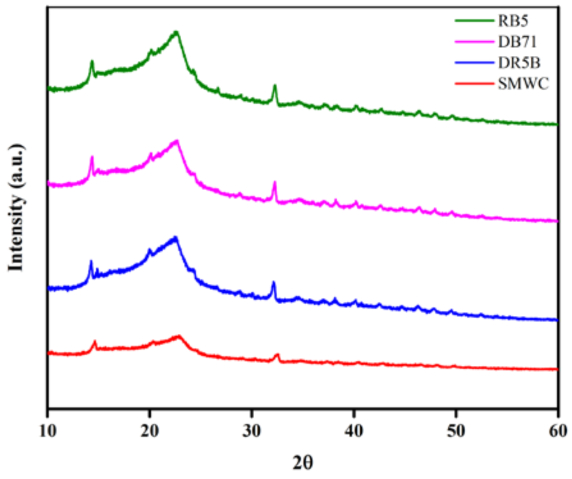

Figure S1. XRD of SMWC absorbing DR5B, DB71, and RB5.

Table S5. Adsorption of dyes from real water samples on SMWC.

Water sample	Dyes	Removal efficiency (%)
Tap water	DR5B	96
	DB71	99
	RB5	99
Lake water	DR5B	96
	DB71	95
	RB5	95
Industrial wastewater	DR5B	80
	DB71	91
	RB5	90
Sea water	DR5B	97
	DB71	95
	RB5	96
